# Relapsing polychondritis following PD-1 blockade diagnosed via 18F-FDG PET/CT and improved by steroid administration: a case report and literature review

**DOI:** 10.3389/fimmu.2025.1619229

**Published:** 2025-08-14

**Authors:** Ruo-Wen Xu, Wen-Bo Dong, Yong-Xu Wang, Yong-Qi Liu, Chen Chen, Wei Wang, Hai-Na Liu, Hong-Yu Jin, Wen-Yang Li

**Affiliations:** ^1^ Respiratory and Critical Care Department, The First Hospital of China Medical University, Shenyang, China; ^2^ First Clinical College, China Medical University, Shenyang, China; ^3^ Department of Rheumatology and lmmunology, The First Affiliated Hospital of China Medical University, Shenyang, China

**Keywords:** cancer, relapsing polychondritis, PD-1 blockade, immune-related adverse events, 18F-FDG PET/CT, ICI-induced RP

## Abstract

**Background:**

Up to 22% of cancer patients treated with immune checkpoint inhibitors (ICIs) can experience immune-related adverse events (irAEs) that mimic rheumatic disease, such as relapsing polychondritis (RP), which is a rare autoimmune disease that mainly manifests as inflammation of airway cartilage.

**Methods:**

We report a case of RP induced by humanized recombinant anti-PD-1 monoclonal antibody therapy (tislelizumab). 18F-Fluorodeoxyglucose positron emission tomography/ computed tomography (18F-FDG PET/CT) contributed to the diagnosis of RP, and methylprednisolone was used to effectively control its progression. We also reviewed 13 publications on drug-induced RP in the context of cancer and analyzed the pathogenesis, ancillary tests, treatment, and prognosis of the cases described therein.

**Results:**

Including our case, 14 drug-related RP cases with a tumor background were analyzed. Patients usually develop related symptoms 3–5 months after initiating medication. The primary tumor involvement sites included the hematological system (5/14, 35.71%), upper digestive tract (4/14, 28.57%), skin (2/14, 14.29%), reproductive system (2/14, 14.29%), bone (1/14,7.14%), and lung (1/ 14, 7.14%).

**Conclusion:**

18F-FDG PET/CT plays a crucial role in diagnosing RP caused by PD-1 monoclonal antibodies. Early detection and the prompt administration of corticosteroids are crucial in effectively controlling the progression of RP, helping to alleviate symptoms and prevent further complications.

## Introduction

Immune checkpoint inhibitors (ICIs) are a novel class of anti-tumor drugs widely utilized in various cancer therapies. However, their use has been associated with an increased incidence of immune-related adverse events (irAEs) that resemble rheumatic diseases (1). Programmed death-1 (PD-1) and its ligand, programmed death-ligand 1 (PD-L1), represent key therapeutic targets in immunotherapy (2, 3). Tislelizumab is a humanized monoclonal IgG4 antibody targeting programmed cell death protein 1 (PD-1). As an immune checkpoint inhibitor (ICI), it blocks the PD-1/PD-L1 interaction, thereby enhancing T-cell-mediated antitumor immunity (4). Relapsing polychondritis (RP) is a rare, systemic inflammatory disorder with an unclear etiology and pathogenesis. The incidence and prevalence of RP remain uncertain, with significant variability in reported data (5).

RP can affect multiple organ systems, primarily targeting cartilage and proteoglycan-rich structures, including the ear, nose, joints, larynx, and skin. Case reports of RP occurring after PD-1 blockade with ICIs have been steadily increasing (6). However, the wide variability in symptoms and clinical manifestations makes diagnosis particularly challenging. Here, we present a case of tislelizumab-induced RP, characterized by lesions in the bilateral costal cartilage and thyroid cartilage, confirmed through 18F-FDG PET/CT imaging. Additionally, we contribute further evidence for drug-induced RP associated with cancer and offer an analysis of its pathogenesis, risk factors, and prognosis, informed by prior case reports.

## Case report

A 56-year-old male was diagnosed with lung cancer (stage ypT1aN0MX) six months ago, with a pathological finding of poorly differentiated squamous cell carcinoma. After two cycles of neoadjuvant chemotherapy combined with immunotherapy (paclitaxel 240 mg and tislelizumab 200 mg on day 1, cisplatin 60 mg on days 1 and 2), the patient underwent a right upper lobe pneumonectomy with mediastinal lymph node dissection. Postoperatively, the same therapy was administered for the first cycle. Subsequently, treatment was switched to single-agent tislelizumab immunotherapy (200 mg) for four cycles, with monthly follow-ups. Three months prior to admission (day 0), the patient developed hoarseness, leading to the suspension of tislelizumab treatment. To determine the cause, blood tests and computed tomography (CT) scans of the lungs were performed. The patient’s white blood cell count was 12.9×10^9^/L, and the C-reactive protein level was elevated at 59 mg/L. CT imaging revealed no significant airway wall thickening; however, tracheoscopy identified slight congestion of the tracheal mucosa. Subsequently, the patient experienced a cough, sputum production, and fever (peak temperature: 38°C), along with shortness of breath, chest tightness, and chest pain exacerbated by physical activity. A repeat lung CT revealed diffuse airway thickening. After 14 days of anti-infective treatment, methylprednisolone (40 mg) was administered for three days to control the fever. Dyspnea resolved, and the fever subsided. One month later, the patient experienced bilateral rib and parasternal pain, worsening cough, and suspected hearing abnormalities. The patient had a history of diabetes mellitus and hypertension.

**Figure 1:**
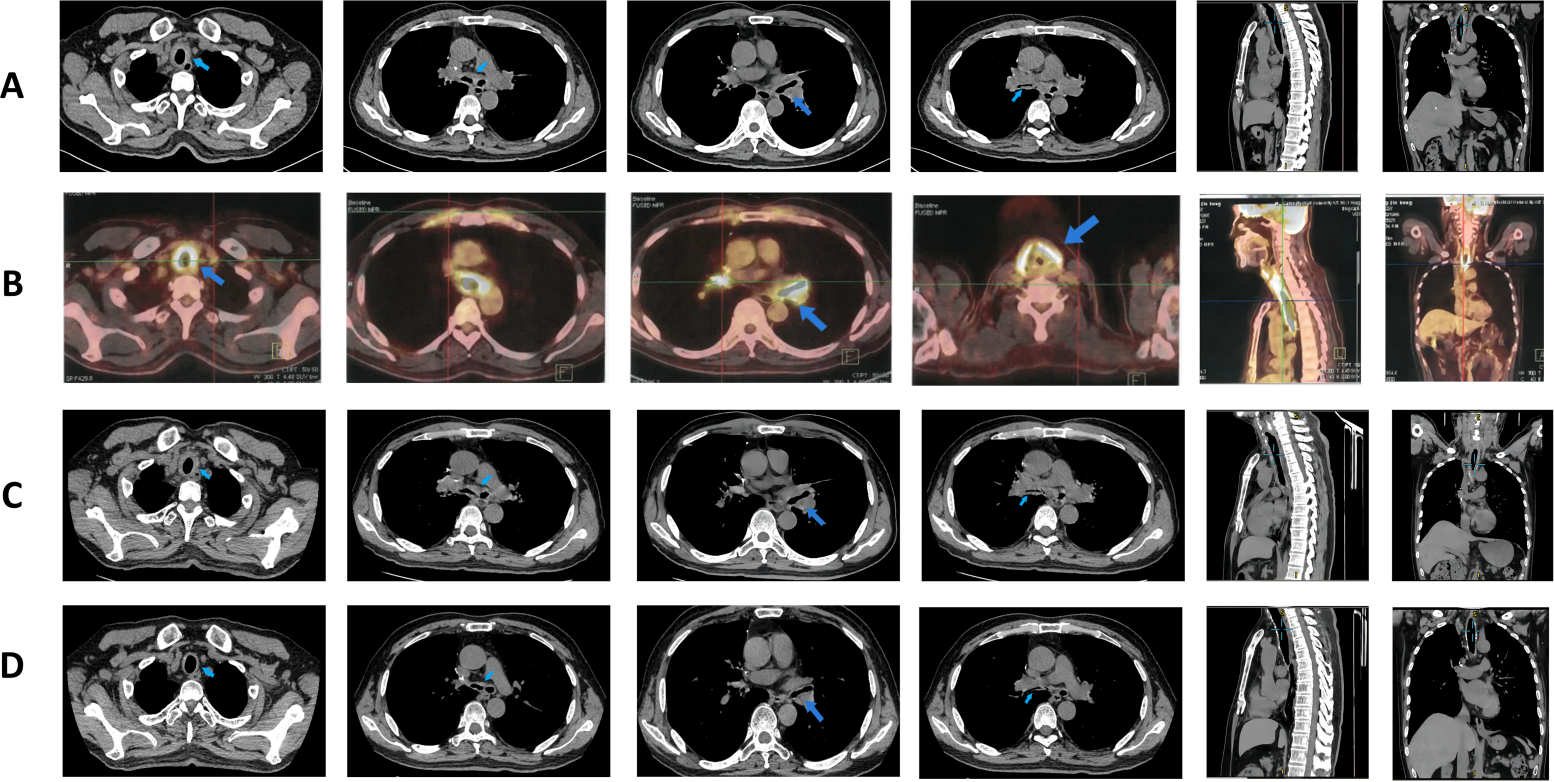
Pulmonary imaging manifestations after disease onset. **(A)** Enhanced CT of the lungs taken on admission day, revealing wall thickening of the trachea and a double main bronchus, along with bronchial stenosis in the middle and lower lobe of the right lung (treatment: piperacillin sodium and tazobactam sodium + levofloxacin). **(B)** ^18^F-FDG PET/CT examination on hospital day 4, revealing diffuse thickening of the trachea, bilateral main bronchi, and parts of the lobar bronchi with diffuse wall thickening; abnormal FDG uptake was also observed in the bilateral costal cartilage and thyroid cartilage areas (treatment: piperacillin sodium and tazobactam sodium + levofloxacin). **(C)** Air tube plain scan 3D-CT taken on hospital day 9, showing no significant changes from before (treatment: levofloxacin + meropenem). **(D)** Lung CT taken at 2 months after admission (treatment: prednisone 40 mg/day).

**Figure 2:**
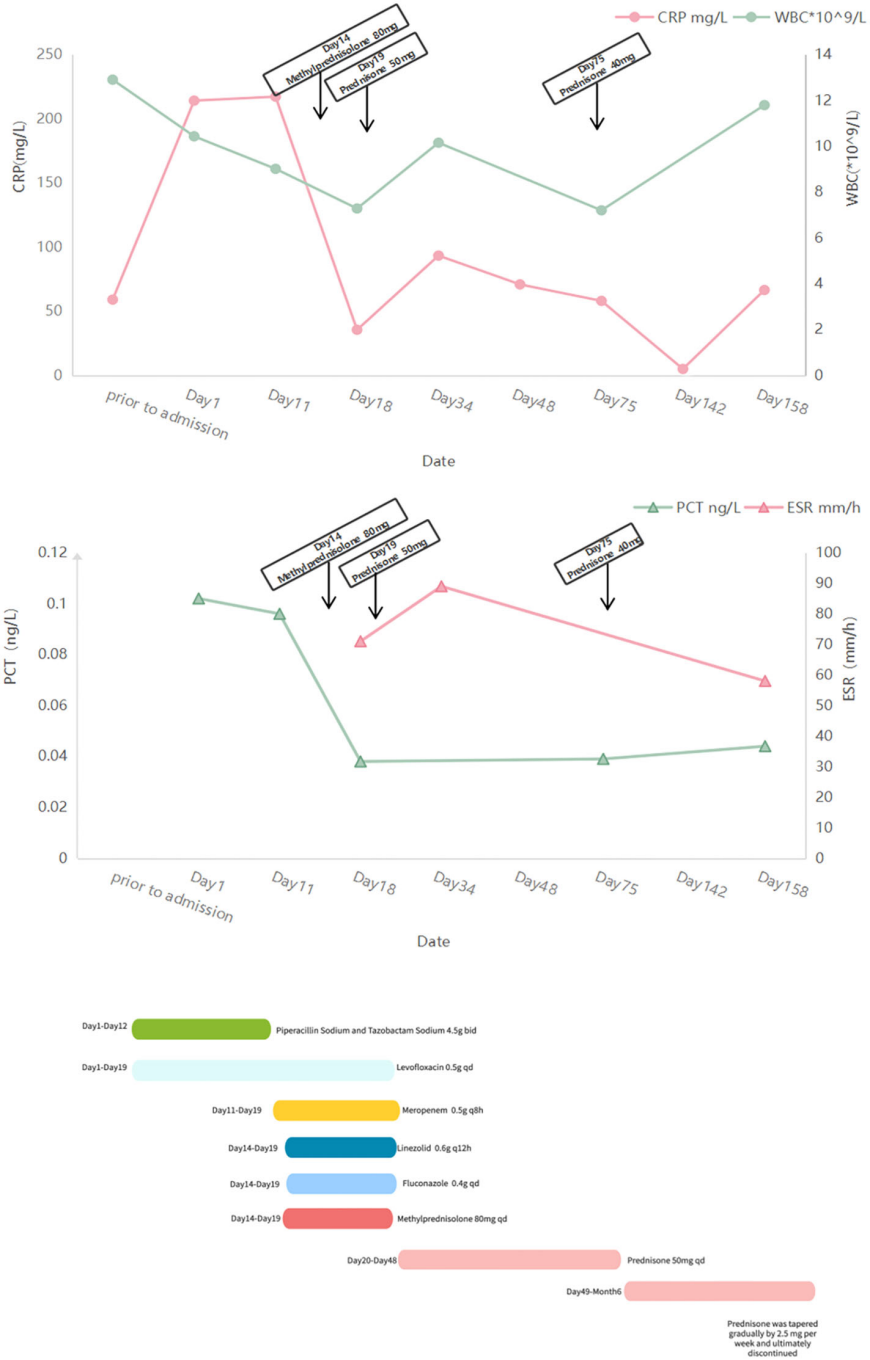
Changes in patient test values (CRP, C-reactive protein; WBC, white blood cell count; PCT, procalcitonin; ESR, sedimentation) Schedule of drug use during hospitalization.

On admission, physical examination revealed a body temperature of 38°C, heart rate of 75 beats/min, respiratory rate of 20 breaths/min, and blood pressure of 134/92 mmHg. Bilateral wheezes were auscultated on lung examination. The remainder of the physical examination was unremarkable. Laboratory test results were as follows: blood gas analysis (room air): pH 7.48, PaCO2–37 mmHg, PaO2–94 mmHg, SaO2 98%, oxygenation index 448 mmHg; C-reactive protein 214 mg/L, procalcitonin 0.102 mg/L, and interleukin-6 76 pg/mL (**Table 1**). Pulmonary function testing revealed: Forced Vital Capacity (FVC): 56.5% predicted; Ratio of Forced Expiratory Volume in 1 second to FVC (FEV1/FVC): 29.92%; Maximal Expiratory Flow at 50% of FVC (MEF50): 7.1% predicted; Maximal Expiratory Flow at 25% of FVC (MEF25): 27.8% predicted; Maximal Mid-Expiratory Flow between 25-75% FVC (MMEF75/25): 8.8% predicted, indicating mixed ventilatory dysfunction, small airway dysfunction, and a 70% ventilatory reserve. The diffusing capacity of the lungs for carbon monoxide (DLCO) test result was 46.8%, indicating a moderate reduction in diffusion capacity. Enhanced lung CT revealed tracheal wall thickening, double main bronchus thickening, and bronchial stenosis in the middle and lower lobes of the right lung (**Figure 1A**). Tracheoscopy revealed diffuse airway mucosal thickening, forming a distinctive “fish-scale” pattern and significant tracheobronchial stenosis. Although histopathological confirmation remains the diagnostic gold standard, biopsy was ultimately deferred due to acute respiratory distress with transient peripheral oxygen desaturation during evaluation, compounded by the patient’s formal refusal of invasive procedures. Given the high anesthesia risk and integrated clinical assessment of disease severity, histopathological confirmation was deemed medically contraindicated. The patient received levofloxacin combined with piperacillin-tazobactam for anti-infective therapy, but his inflammatory markers showed no significant improvement. An 18F-FDG PET/CT scan revealed diffuse thickening of the trachea and lobar bronchial walls, as well as bilateral main bronchi. Abnormal FDG uptake was also detected in the bilateral costal cartilage (SUV max 4.1) and thyroid cartilage (SUV max 8.9) (**Figure 1B**). Next-generation sequencing of sputum identified fungi, viruses, and bacteria (**Table 2**). Following treatment with levofloxacin and piperacillin-tazobactam, a plain 3D CT scan of the trachea showed no significant changes compared to the previous scan (**Figure 1C**). Consequently, the anti-infective treatment plan was adjusted. On hospital day 14, meropenem, linezolid, and fluconazole were administered for anti-infective therapy in combination with methylprednisolone (80 mg/day) for anti-inflammatory treatment. (see **Figure 2** for the detailed drug schedule). After six days, his inflammatory markers significantly improved: C-reactive protein decreased to 35.5 mg/L, procalcitonin to 0.038 ng/L, and erythrocyte sedimentation rate to 89 mm/h. Following active treatment, the patient’s respiratory symptoms improved, with no fever recurrence. Based on the patient’s medical history, clinical presentation, and imaging findings, a diagnosis of tislelizumab-induced RP was made.

**Table 1:** Laboratory test results and reference ranges. (The first day of hospitalization).

Category	Test Item	Result	Reference Range
**Routine Blood Examination**	White blood cell (10^9^/L)	10.43	3.5–9.5
	Hemoglobin (g/L)	139	130–175
	Platelets (10^9^/L)	208	125–350
**Coagulation Profile**	PT (s)	12.1	9.0–13.0
	INR	1.06	0.8–1.2
	APTT (s)	30.5	23.3–32.5
	FIB (g/L)	8.45	2.00–4.00
	TT (s)	15.50	14.00–21.00
	D-Dimer (μg/mL)	1.49	0.00–0.50
**Inflammatory Markers**	CRP (mg/L)	214	0.00–6.00
	PCT (ng/mL)	0.102	0.000–0.046
	IL-6 (pg/mL)	76	1.50–7.00

PT, prothrombin time; INR, international normalized ratio; APTT, activated partial thromboplastin time; FIB, fibrinogen; TT, thrombin time; CRP, C-reactive protein; PCT, procalcitonin; IL-6, interleukin-6.

**Table 2:** Next-generation sequencing of sputum revealing the presence of fungi, viruses, and bacteria (tuberculosis was not found).

Category	Pathogen	Reads
Fungus	Candida albicans	58
Virus	Human alphaherpesvirus 1	47
	Human herpesvirus type 5	24
	Human betaherpesvirus 7	6
Background bacteria	Corynebacterium argentoratense	12785

In this case, differential diagnoses included paraneoplastic syndrome, bronchial asthma, tracheobronchial amyloidosis, stenosis secondary to extrinsic compression, tracheal malignancy, pulmonary infection and granulomatosis with polyangiitis (GPA). PET-CT imaging demonstrated no evidence of malignant transformation at the tracheal site, with inflammatory changes confined to chondritis. This effectively excluded cancer progression as the etiology of RP. All serum tumor markers were negative in this patient. Blood cultures remained negative after 5 days of incubation. No saddle-nose or cauliflower ear deformities were observed. All rheumatologic antibodies tested negative. The absence of clinical or biochemical improvement following comprehensive antimicrobial therapy effectively excluded infectious causes. The patient demonstrated a negative bronchodilator response, and bronchoscopy revealed no evidence of extrinsic compression, effectively ruled out bronchial asthma, and externally compressed airway narrowing. Tracheobronchial amyloidosis was definitively excluded based on bronchoscopic findings revealing no circumferential wall thickening involving the membranous portion, coupled with CT imaging demonstrating absence of tram-track calcifications, and unresponsiveness to corticosteroid therapy. GPA was definitively excluded based on negative ANCA serology, absence of renal involvement, no evidence of bony destruction, and lack of necrotizing granulomas on bronchoscopy.

Although RP was diagnosed without histological confirmation, the patient’s dyspnea with wheezing, PET-CT demonstrating increased metabolic activity at three distinct anatomical sites (thyroid cartilage, tracheal rings, and costal cartilages) alongside diffuse tracheal thickening, bronchoscopic findings of diffuse mucosal thickening and tracheal stenosis—coupled with poor response to antibiotics but with a clinically significant response to steroid therapy—collectively fulfilled the Damiani and Levine diagnostic criteria for RP. Consequently, after exclusion of alternative etiologies, ICI-induced RP was strongly suspected given its onset 3 months post-immunotherapy. The patient was discharged on oral prednisone (50 mg daily), with instructions to taper the dose gradually by 2.5 mg per week.

**Figure 3:**
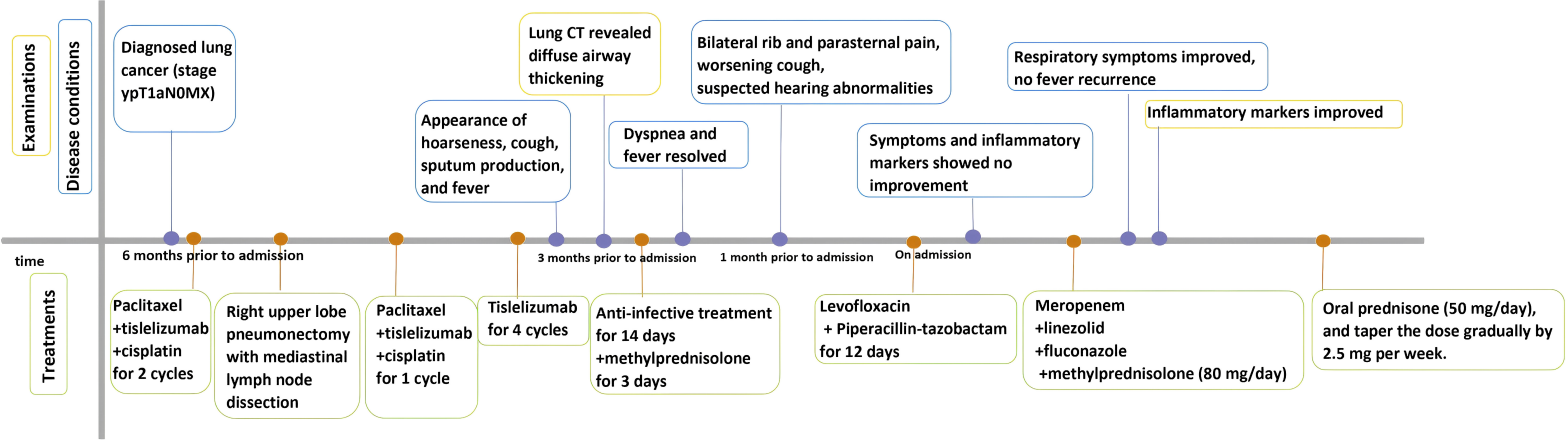
The timeline of the patient’s symptom, diagnosis and treatments.

After discharge, the patient underwent monthly lung CT scans (**Figure 1D**), which showed significant improvement, confirming the efficacy of prednisone therapy. The patient continued regular blood tests and CT scans. The follow-up examination results were shown in **Figures 2**, **3**. One month later, his chest tightness and shortness of breath had significantly improved. Auscultation revealed dry wheezes in both lungs, though they were notably reduced compared to prior assessments. Two months later, the patient’s prednisone dose was reduced to 40 mg/day. Blood tests showed pH 7.424, PaCO2 45.2 mmHg, PaO2 66.2 mmHg, SaO2 89.1%, and an oxygenation index of 315 mmHg. The inflammatory markers had significantly improved. Pulmonary function tests indicated mixed ventilation impairment and small airway dysfunction: FVC 46.4 L, FEV1/FVC 40.25%, MEF50 8.8%, MEF25 18.6%, MMEF75/25 11.1%. The patient continued oral prednisone therapy. An 18F-FDG PET/CT showed that the SUV metabolic value of the trachea, bilateral main bronchi, and some lobar bronchial tubes decreased from 9.2 to 4.2, indicating significant improvement in respiratory symptoms. Five months later, when his prednisolone dose had been gradually reduced to 17.5 mg/day, the patient developed costal cartilage and sternal pain, possibly due to dose reduction. The patient was maintained on oral prednisone 17.5 mg daily for one month, followed by a protocol-guided taper until complete discontinuation, without disease recurrence. The patient has maintained regular follow-up with our team. At the one-year post-discharge evaluation, all examinations demonstrated favorable results: prednisone therapy has been discontinued, there was no evidence of cancer progression, and the patient reported only cough with sputum occasionally.

Regrettably, due to delayed diagnosis and resource constraints in our laboratory setting, we were unable to perform anti-collagen type II antibodies testing for this patient. This assessment is planned for completion at the next scheduled follow-up visit.

## Literature review

**Figure 4:**
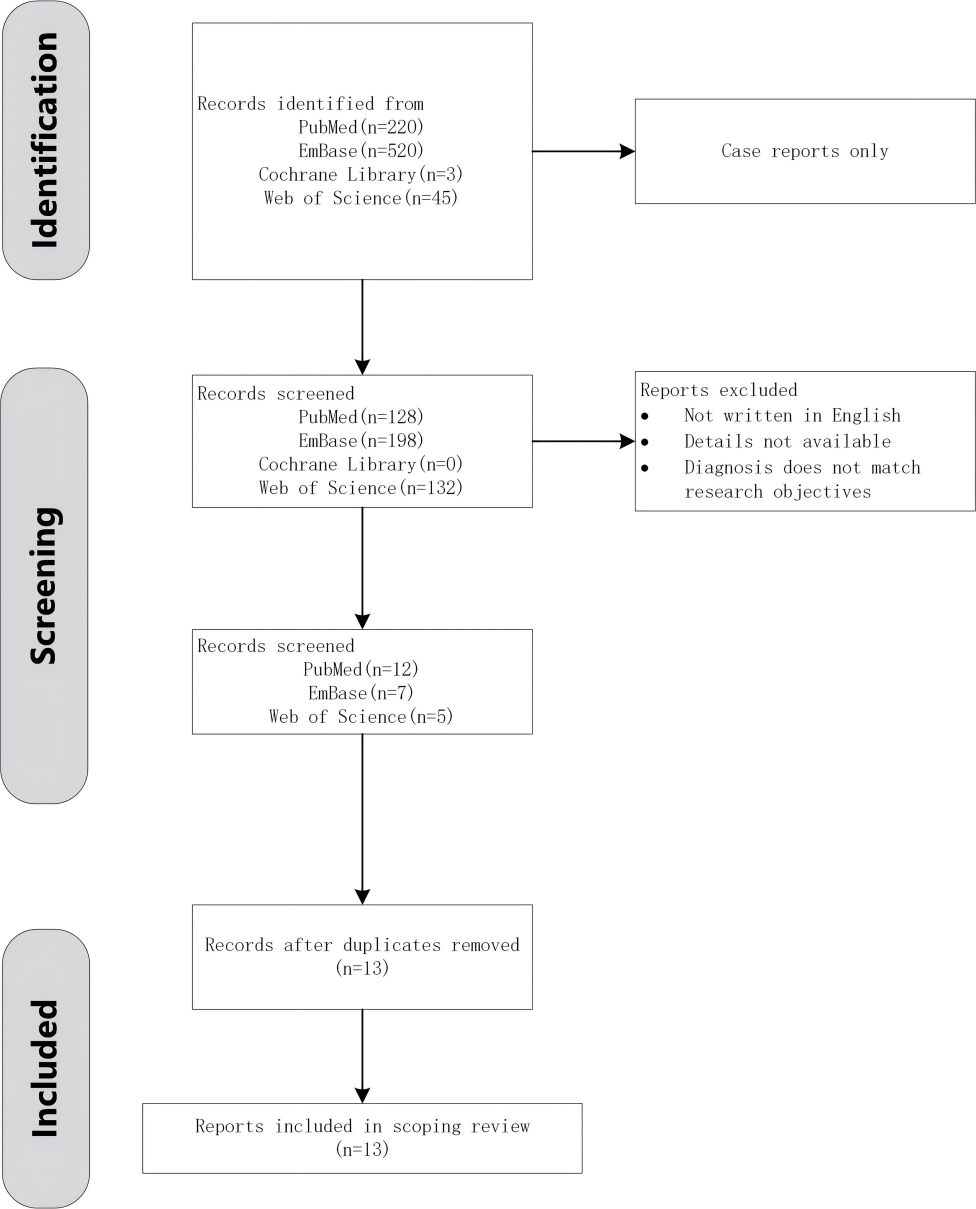
Flow chart of bibliographic database retrieval.

We searched the PubMed, Web of Science, Cochrane, and Embase databases for published articles on cancer associated with RP (**Figure 4**). The search terms included “relapsing polychondritis”,”polychondritides, relapsing”, “relapsing polychondritides”, “polychondritis, chronic atrophic”, “atrophic polychondritides, chronic”, “atrophic polychondritis, chronic”, “chronic atrophic polychondritides”, “chronic atrophic polychondritis”,”polychondritides, chronic atrophic”, and “neoplasms”, “tumor”, “neoplasm”, “tumors”,”neoplasia”, “neoplasias”, “cancer”, “cancers”, “malignant neoplasm”, “malignancy”, “malignancies”, “malignant neoplasms”, “neoplasm, malignant”, “neoplasms, malignant”, “benign neoplasms”, “benign neoplasm”, “neoplasms, benign”, “neoplasm, benign”; these were used in various combinations, with or without the Boolean operator ‘AND’ to combine them. Criteria for inclusion included Patients with definitively diagnosed relapsing polychondritis (RP) and concomitant malignancy. Exclusion criteria included studies not written in English, incomplete/unavailable primary data, reports not aligned with study objectives (e.g., drug-induced RP without malignancy association). A thorough manual review of the bibliographies of the selected articles yielded 13 relevant articles, all reporting cases of RP secondary to medication use following cancer treatment (7–19).

Specifically, airway involvement (tracheobronchial system, 7/14, 50.00%) and auricular chondritis (5/14, 35.71%) predominated, whereas articular, ocular, and cutaneous manifestations each occurred in 14.29% of cases (2/14). Notably, no neurological involvement was observed. No cases demonstrated cardiovascular involvement, neurological manifestations, or hematological disorders. Including our case, a total of 14 cases of RP following tumor treatment have been reported (**Table 3**). Among these, 11 cases were reported following treatment with biologics, including 7 cases after PD-1 inhibitor use and 1 case after chemoradiotherapy. Patients typically developed associated symptoms 3 to 5 months after medication use. Our case represents the first report of drug-associated RP in the context of lung cancer, with symptoms appearing 2 months after medication initiation. In the 14 patients, tumor sites primarily included the blood system (5/14, 35.71%), upper digestive tract (4/14, 28.57%), skin (2/14, 14.29%), reproductive system (2/14, 14.29%), bone (1/14, 7.14%), and lung (1/14, 7.14%).

**Table 3:** Demographic and clinical characteristics of patients with drug-induced recurrent polychondritis in the context of cancer.

Case	Patient A/S	Preexisting tumor	Drugs used	Time from medication to onset of recurrent polychondritis	Affected part of the respiratory tract	Affected part of the extra- respiratory system	C-reactive protein	Anti-CII	Treatment	Discontinue medication or not	Prognosis	Country	Reference
1	72/M	Mandibular carcinoma	Nivolumab	23 months	+	−	Increase	Unknown	ICS	Yes	No deterioration	Japan	Ogimoto et al. (7)
2	72/M	Squamous cell carcinoma of hypopharynx	Nivolumab	4 months	+	−	Increase	+	Intravenous hydrocortisone + oral prednisone	Yes	Improved	Japan	Kuba et al. (8)
3	75/M	Prostate cancer	Goserrelinomab	6 months	−	+ (Skin)	Increase	Unknown	Indomethacin + Prednisone	Yes	Death (cancer progression)	France	Labarthe et al. (9)
4	49/M	Squamous cell carcinoma of the lower lip	Pembrolizumab	4 months	−	+ (Ear)	Increase	Unknown	Stop Pembrolizumab and replace it with Paclitaxel	Yes	Death (cancer progression)	Japan	Mutoh et al. (10)
5	68/M	Laryngeal cancer + esophageal cancer	5-FU+Cisplatin+Nivolumab	5 months	+	−	Increase	+	Prednisone	Yes	Improved	Japan	Asoh et al. (11)
6	71/M	Squamous cell carcinoma of hypopharynx	Cisplatin + Radiotherapy +Nivolumab	5 months	+	−	Increase	+	Prednisone	Unknown	Improved	Japan	Someya et al. (12)
7	66/M	Hypopharyngeal carcinoma	Nivolumab	12 months	+	+ (Chest)	Increase	+	Steroid and methotrexate reduction after recurrence, plus Tocilizumab	Unknown	Improved	Japan	Hamada-Ode, et al. (13)
8	60/F	Splenic non-Hodgkin lymphoma	Cyclophosphamide, doxorubicin hydrochloride, vincristine sulfate, prednisolone 3 series of treatment + local radiotherapy	Unknown	−	+ (Ear,Eye, Auditory system, Skin)	Increase	Unknown	Loxoprofen sodium and diclofenac sodium did not improve, changed to prednisone	Unknown	Improved	Japan	Yanagi et al. (14)
9	41/M	Hodgkin’s lymphoma	MOPP treatment did not work and ABVD was changed. This was followed by mantle irradiation and chemotherapy cycles including lomustine and vincristine.	Unknown	−	+ (Ear)	Normal	Unknown	Prednisone + Amoxicillin–Clavulanate potassium	Unknown	Blood deterioration	America	Robert et al. (15)
10	69/M	Adenocarcinoma of prostate	LH-RH analogue Buserrelin and pure antiandrogen bicalutamide (Casodex)	5 months	−	+ (Ear)	Increase	Unknown	Prednisone + Azathioprine + Methotrexate	Yes	Improved	Unknown	Rozin et al. (16)
11	72/M	RS3PE and MDS, refractory anemia subtypes	Occures when prednisone is reduced	Unknown	−	+ (Joints, Nose)	Increase	Unknown	Prednisone	Yes	Death (heart failure)	Israel	Manganelli et al. (17)
12	70/M	Esophageal squamous cell carcinoma	Docetaxel + cisplatin + 5-FU	Unknown	+	+ (Auditory system)		Unknown	Prednisone	Yes	Improved	Japan	Mase et al. (18)
13	57/M	Myeloproliferative tumor	Hydroxyurea	Unknown	−	+ (Ear, Joints, Eye)		Unknown	Ruxolitinib	Yes	Unknown	Turkey	Yavaşoğlu et al. (19)
14 (our case)	56/M	Lung malignant tumor	Tislelizumab	5 months	+	+ (Chest, Auditory system)	Increase	Unknown	Methylprednisolone	Yes	Improved	China	Xu et al.

A/S, age/sex; M, male; F, female; anti-CII, Anti-type II collagen antibodies; ICS, inhaled corticosteroid; 5-FU, 5-fluorouracil; MOPP, monoaxial oriented polypropylene; ABVD, adriamycin bleomycin vinblastine dacarbazine; LH-RH, luteinizing hormone releasing hormone.;+, Positive; -, Negative.

In terms of diagnoses, lung CT revealed tracheal and bronchial involvement in 46.67% of cases, with irregular tracheal wall thickening observed in 80%. Additionally, 35.71% (5/14) of patients underwent bronchoscopy, while 14.29% (2/14) underwent laryngoscopy. During endoscopic examination, 71.43% (5/7) exhibited redness and swelling of the tracheobronchial mucosa, while 28.57% (2/7) showed the disappearance of cartilage rings. Finally, 50% (7/14) of patients underwent an 18F-FDG PET/CT examination, with 85.71% (6/7) showing increased uptake values at lesion sites.

Regarding laboratory examinations, RP lacks a blood marker with high specificity. In standard laboratory tests, 85.71% (13/14) of cases showed elevated C-reactive protein levels, 42.86% (6/14) showed increased ESR, 28.57% (4/14) exhibited decreased hemoglobin, 21.43% (3/14) demonstrated elevated white blood cell counts, and 7.14% (1/14) showed both increased and decreased platelet levels. Four patients tested positive for anti-type II collagen antibodies, one for antinuclear antibodies, one had elevated urinary protein levels, one showed elevated C3 and C4 levels, one tested positive for IgG4, and one exhibited high level of MMPase 3.

Regarding treatment, the 14 summarized cases suggest that RP in the context of tumor treatment has a better prognosis; this may be due to a relatively clear etiology, facilitating straightforward intervention. Symptoms in two cases resolved within 2 months of drug withdrawal (10, 19). The initial prednisone treatment dose ranged from 30 to 60 mg daily, with most patients observing symptom improvement within 1 week. After 2 weeks, the airway wall typically demonstrated partial or total recovery. However, four patients experienced recurring symptoms during dose reduction. Among these, two patients who initially received immunosuppressive agents relapsed upon dosage reduction but improved after reintroduction of the agents (13, 17). The other two cases experienced recurrence due to overly rapid or excessive dose reductions. One case improved after returning to the original dose (8), while one improved after adding inhaled glucocorticoids for recurrent respiratory symptoms (7).

Regarding prognoses, treatment resulted in noticeable improvement in RP symptoms for 78.57% (11/14) of patients. Among these, three experienced improvements; However, two ultimately died from cancer progression (9, 10), and one died from heart failure (17). One patient exhibited no significant changes in symptoms (7), another experienced deterioration in blood indicators (15).

## Discussion

To our knowledge, this case represents the first report of tislelizumab-induced RP with lesions in the bilateral costal cartilage and thyroid cartilage. Enhanced understanding of drug-induced RP will improve patient care and aid in predicting the risk of developing severe toxicities associated with ICIs.

Due to the varying pharmacological effects of antitumor drugs, differing routes of administration, and patient conditions, adverse reactions differ significantly and are often challenging to recognize early clinically (20, 21). Immune checkpoint inhibitors (ICIs) can cause immune-related adverse events (irAEs), believed to result from immune system activation, manifesting as diverse symptoms resembling autoimmune disorders (22). Mechanistically, both ICIs and irAEs involve the bystander effect of activated T cells (23, 24). While ICIs elicit specific anti-tumor responses, this T lymphocyte hyperactivation can also cause “on-target” side effects in normal tissues IrAEs can manifest widely in severity and type, and they were frequently reported (25). Among reported irAEs, rheumatic manifestations encompass arthralgia, arthritis, myalgia, myositis, vasculitis, sicca syndrome, scleroderma, and systemic lupus erythematosus (25, 26). Tislelizumab is a humanized monoclonal antibody exhibiting high affinity and specificity for PD-1. It was specifically engineered to minimize FcγR binding on macrophages, abrogating antibody-dependent phagocytosis—a potential mechanism underlying T-cell clearance and resistance to anti-PD-1 therapy (27). We propose that the lower incidence of tislelizumab-associated RP, compared with other PD-1 inhibitors, may be attributed to its FcγR binding affinity, target engagement profile, and divergent immune effector functions (28). Recent findings also highlight the importance of Fc-FcγR interactions for the *in vivo* activities mediated by immunomodulatory Abs (29). However, as immune checkpoint inhibitor-induced RP remains limited to rare individual case reports, the precise underlying mechanisms warrant further investigation. Analysis of the 14 screened case reports indicates that respiratory tract involvement is more frequently observed in PD-1 inhibitor-induced RP. However, given that our case represents the first reported instance of tislelizumab-induced RP, the limited sample size was unable to make a meaningful comparison of clinical manifestations between tislelizumab and other PD-1 inhibitors. Multicenter collaborations and validation with larger clinical cohorts are warranted for definitive conclusions.

The safety of tislelizumab, as an ICI, was initially evaluated in the RATIONALE 304 trial, which reported serious adverse events primarily including pneumonia and anemia (2, 27, 30, 31). Recent studies have identified irAEs induced by tislelizumab, either alone or in combination with other drugs, with various manifestations, including severe thyrotoxicosis (32), hypersensitivity reactions (33), and pancytopenia (34).

RP is a rare inflammatory disease with an unknown etiology and pathogenesis that can affect multiple systems (6), particularly ears, nose, and tracheobronchial cartilage (35). Classic clinical manifestations include cauliflower ear, saddle nose, and ocular inflammation. Respiratory tract involvement can include tracheomalacia, bronchomalacia, thickening of the gas duct wall, and subglottic stenosis (36). RP primarily affects hyaline and elastic cartilage following treatment with PD-1 inhibitors. At disease onset, tracheal and bronchial lesions are less common than auricular lesions, which are the hallmark feature of RP (35). In ICI-induced RP, the most commonly involved sites are: auricular cartilage, respiratory tract, nasal cartilage, joints, et al. Compared to idiopathic RP, where auricular and nasal cartilage involvement is most typical early in the disease, ICI-induced RP shows a notably higher frequency of airway involvement and often presents more acutely (10). As reported in some cases, airway symptoms are the initial or dominant presentation in ICI-induced RP, whereas idiopathic RP tends to evolve more gradually with a broader systemic pattern (37). We compared the frequency of symptoms between idiopathic RP and ICI-induced RP (**Table 4**), but due to the limited number of reported cases of ICI-induced RP in the literature, the frequencies should be interpreted with caution.

**Table 4:** Comparative organ involvement in idiopathic RP vs. ICI-induced RP.

Idiopathic RP	Cumulative frequency during follow-up	ICI-induced RP	Cumulative frequency during follow-up
Chondritis of the ear	70 to 95%	Laryngotracheobronchial chondritis	50%
Chondritis of the nose	35 to 63%	Chondritis of the ear	36%
Sternocostal chondritis	44 to 65%	Audio-vestibular involvement	21%
Laryngotracheobronchial chondritis	21 to 56%	Sternocostal chondritis	14%
Inflammatory joint disease	52 to 85%	Eye involvement	14%
Eye involvement	44 to 65%	Inflammatory joint disease	14%
Audio-vestibular involvement	19 to 46%	Skin involvement	14%
Skin involvement	17 to 46%	Chondritis of the nose	7%
Valvular involvement	6 to 27%		
Central neurological manifestations	5 to 9%		
Myelodysplasia	6 to 9%		

The pathogenesis remains unclear but is presumed to involve autoimmune responses against type II collagen (38). It is presumed that specific autoantibodies are produced against type II collagen, matrilin-1, and cartilage oligomeric matrix protein (COMP), all components of hyaline cartilage (39–41). Anti-type II collagen antibodies correlate with disease activity but are detectable in only 30–60% of patients Consequently, early diagnosis is challenging due to nonspecific symptoms. Currently, three diagnostic criteria are recognized: McAdam (42), Damiani (43), and Michet-Hughes (44). In this case, chondritis occurred at three distinct sites (thyroid cartilage, tracheal cartilage, costal cartilage) with good response to steroids, thus fulfilling the Damiani and Levine criteria (43).

In terms of imaging for RP, 18F-FDG PET/CT and bronchoscopy are valuable for diagnosis, evaluating disease activity, and predicting treatment responses, especially in patients with airway involvement.

18F-FDG PET/CT imaging may serve as a sensitive method for identifying the development and severity of irAEs, which typically present as new non-neoplastic lesions with increased FDG accumulation following ICI treatment (45–48). Studies have demonstrated that 18F-FDG PET/CT is effective in assessing the activity and extent of RP, evaluating treatment response, and detecting recurrence (49, 50), while also serving as a valuable tool for therapeutic monitoring during follow-up evaluations, albeit with recognized limitations in its clinical utility (51, 52).

Nevertheless, clinical limitations persist in its practical application. The critical role of PET/CT in diagnosing RP has been well-established. Whole-body ^18^F-FDG PET/CT visualizes inflammatory and infectious lesions through tracer uptake in both malignant and inflammatory cells (53). Therefore, PET can assist in diagnosing RP, but it requires combining the history of specific drug exposure, other laboratory test results, and response to corticosteroid therapy, so as to comprehensively determine the cause of RP.

Typical PET/CT findings for involved airways show symmetrically increased FDG uptake and thickening of the laryngo-tracheobronchial wall, with a potential decrease in radioactivity following effective glucocorticoid treatment (50). This method is especially valuable when biopsies fail to yield a diagnosis (46).

In comparison to 18F-FDG PET/CT, bronchoscopy has a higher detection rate for tracheal abnormalities but a lower detection rate for peripheral airway issues (54). A prior study reported significant FDG accumulation in the airways of RP patients, even in the absence of respiratory function and CT abnormalities, indicating that asymptomatic chondritis can be detected using 18F-FDG PET/CT (55). Although SUVmax is a commonly used metabolic parameter, its prognostic value remains debated (56, 57), with some researchers proposing that SUVmean may be more indicative (58). In our patient, no respiratory symptoms were present prior to starting tislelizumab. The patient presented only with atypical symptoms, such as chest tightness and shortness of breath. Although cartilage inflammation was not pathologically confirmed, tracheoscopy, lung CT, and 18F-FDG PET/CT imaging—combined with the discontinuation of tislelizumab and the addition of glucocorticoids—significantly improved symptoms. A pulmonary CT examination conducted one month later revealed that the airway wall was significantly thinner, suggesting that early glucocorticoid intervention can improve both symptoms and prognosis. This case met the diagnostic criteria established by Damiani and Levine (43) and can be clinically diagnosed as RP induced by tislelizumab. Additionally, this case demonstrated that the combination of bronchoscopy and PET/CT may provide a more reliable and comprehensive approach for evaluating the extent of inflammation in RP.

Inflammatory indicators can assist in the diagnosis of RP and the evaluation of treatment efficacy. Liu et al. (59) found that, elevated CRP, IL-6, ESR, complement 3, platelet-to-lymphocyte, neutrophil-to-lymphocyte, and C-reactive protein-to-albumin ratios correlate positively with disease activity. These markers are typically elevated and rapidly decline with treatment (60). Similarly, this case presented elevated CRP, IL-6, and procalcitonin, while the Procalcitonin (PCT) value (0.102 ng/mL) was only mildly elevated. It has been recognized that PCT is a biomarker that tends to rise significantly in response to microbial pathogens. However, PCT is less sensitive for non-infectious inflammatory conditions and typically does not exhibit a significant elevation in cases of autoimmune or drug-induced inflammation, such as in the case of ICI-induced RP. In the current case, while PCT levels were mildly elevated (0.102 ng/mL), this value is generally considered low and would not support the diagnosis of a bacterial infection. Additionally, the levels of CRP and IL-6, which are more responsive to systemic inflammation and immune activation, were significantly elevated, further supporting the likelihood of an inflammatory response related to RP rather than infection. Thus, infection was considered less likely given the low PCT value in combination with the overall clinical picture and laboratory findings.

Although anti-type II collagen antibodies exhibit some specificity, only 30–60% of RP patients test positive for these antibodies (61).Genetic alterations, such as HLA typing and UBA1 somatic mutations, have been identified in RP patients and may also serve as potential biomarkers (62).

Due to the absence of evidence-based treatment guidelines, the management of RP is primarily empirical. There is no specific or systematic treatment protocol; Glucocorticoids and nonsteroidal anti-inflammatory drugs (NSAIDs) are effective in controlling disease progression. The dosage of corticosteroids varies among individuals and is contingent upon disease severity. For nonvital organ involvement (e.g., ear, nose, joints), prednisone (30–60 mg/day) is effective, followed by gradual tapering to prevent recurrence (63). For severe or life-threatening involvement, prednisone (1 mg/kg) plus cyclophosphamide (2 mg/kg/day) is recommended; lack of improvement within one month warrants second-line agents like methotrexate. The optimal duration of treatment for immune checkpoint inhibitor (ICI)-induced RP remains uncertain, with relapses frequently occurring if corticosteroid tapering is too rapid—highlighting the need for a cautious and gradual reduction strategy. The French recommendations suggest that for laryngeal and tracheobronchial chondritis, initial corticosteroid therapy is 0.5 to 1 mg/kg/day of prednisone-equivalent, not exceeding 60 to 70 mg/day for at least 3 weeks depending on severity and clinical course, followed by a tapering dose with the aim of reaching a dosage of less than or equal to 15 mg/day at 3 months and less than or equal to 10 mg/day at 6 months. Infusions of methylprednisolone (250–1000 mg/day for 1–3 days) may be used depending on severity. Inhaled corticosteroid therapy may be used in combination (64). Additionally, decisions regarding the duration of steroid therapy and restarting ICI therapy should be guided by the severity of RP and the patient’s prior clinical response to immunotherapy. Interestingly, the manifestations seem to resolve after discontinuation of ICI treatment and relapse after re-treatment (10). A pulse dose of hydrocortisone (1000 mg daily) has been reported to alleviate acute respiratory obstruction (65). The combination of glucocorticoids with methotrexate, cyclophosphamide, azathioprine, cyclosporin A, and other immunosuppressive agents can enhance disease control, facilitate glucocorticoid reduction, and minimize side effects (66). Conversely, biologic agents should be reserved for patients who demonstrate inadequate responses to conventional DMARDs or develop significant intolerance to prior immunosuppressive regimens. Among biologics, TNFα inhibitors currently represent the first-line option for RP (67). Recurrence is common with premature or rapid steroid withdrawal. Treatment parallels that for high-grade irAEs: cautious corticosteroid tapering prevents recurrence, while severe or steroid-refractory cases require additional immunosuppression (68). The optimal treatment duration for drug-induced RP remains unclear. In cases where combined immunosuppressive therapy fails or in severe disease, biologic agents, particularly TNFα inhibitors (e.g., infliximab, adalimumab), are commonly employed (67).

Clinicians should recognize that RP can manifest as an irAE. Due to its atypical symptoms, the absence of specific diagnostic indicators, and high examination costs, diagnosis often relies on clinical symptoms and can be delayed. Patients presenting with airway involvement as the initial symptom may experience a insidious onset, rapid disease progression, severe respiratory symptoms, and a poor prognosis, often leading to life-threatening complications. The methods for diagnosing and differentiating primary from secondary chondritis, as along with the optimal systemic medication strategies, remain topics of ongoing discussion. Given the multi-organ involvement in RP, treatment should be managed by a multidisciplinary team of physicians who are well-versed in its pathogenesis, clinical manifestations, potential symptoms, diagnostic examinations, and pharmacological therapies. Early detection, diagnosis, and treatment are essential to prevent the irreversible progression of tracheal and bronchial chondritis, alleviate patient suffering, prolong survival, and enhance quality of life.

## Data Availability

The raw data supporting the conclusions of this article will be made available by the authors, without undue reservation.
